# Heterotrimeric G-Protein γ Subunit CsGG3.2 Positively Regulates the Expression of *CBF* Genes and Chilling Tolerance in Cucumber

**DOI:** 10.3389/fpls.2018.00488

**Published:** 2018-04-17

**Authors:** Longqiang Bai, Yumei Liu, Ying Mu, Ali Anwar, Chaoxing He, Yan Yan, Yansu Li, Xianchang Yu

**Affiliations:** ^1^Institute of Vegetables and Flowers, Chinese Academy of Agricultural Sciences, Beijing, China; ^2^College of Agricultural and Biological Engineering, Heze University, Heze, China

**Keywords:** G-protein γ subunit, CsGG3, cucumber, cold stress, *CBF* genes

## Abstract

Heterotrimeric guanine nucleotide-binding proteins (G proteins) composed of alpha (Gα), beta (Gβ), and gamma (Gγ) subunits are central signal transducers mediating the cellular response to multiple stimuli, such as cold, in eukaryotes. Plant Gγ subunits, divided into A, B, and C three structurally distinct types, provide proper cellular localization and functional specificity to the heterotrimer complex. Here, we demonstrate that a type C Gγ subunit CsGG3.2 is involved in the regulation of the *CBF* regulon and plant tolerance to cold stresses in cucumber (*Cucumis sativus* L.). We showed that *CsGG3.2* transcript abundance was positively induced by cold treatments. Transgenic cucumber plants (T1) constitutively over-expressing *CsGG3.2* exhibits tolerance to chilling conditions and increased expression of *CBF* genes and their regulon. Antioxidative enzymes, i.e., superoxide dismutase, catalase, peroxidase, and glutathione reductase activities increased in cold-stressed transgenic plants. The reactive oxygen species, oxygen free radical and H_2_O_2_, production, as well as membrane lipid peroxidation (MDA) production decreased in transgenic plants, suggesting a better antioxidant system to cope the oxidative-damages caused by cold stress. These findings provide evidence for a critical role of CsGG3.2 in mediating cold signal transduction in plant cells.

## Introduction

Cold stress, which includes chilling (<20°C) and/or freezing (<0°C) temperatures, adversely affects the growth and development of crops, and results in heavy economic losses (Chinnusamy et al., 2007). Plants exhibit an increase in chilling tolerance after being exposed to low non-freezing temperatures, which is known as cold acclimation (Medina et al., 2011). During cold acclimation, genes coding CBFs (C-repeat binding factors), HSFC1 (heat shock transcription factor C 1), ZAT12 (zinc transporter of *Arabidopsis thaliana* 12), and CZF1 (CCCH-type zinc finger 1) are induced, and in turn regulate downstream cold responsive gene (COR) expression making changes in metabolism and physiological processes (Jia et al., 2016; Zhao et al., 2016). These include the changes in the activity of antioxidant enzymes, such as ascorbate peroxidase (APX), superoxide dismutase (SOD), and catalase (CAT), which contributes to increased freezing tolerance (Streb et al., 2002; Janda et al., 2003; Zhang et al., 2008). CBFs, also known as dehydration-responsive element (DRE) binding factor 1 (DREB1), are key transcription factors involved in cold response (Jia et al., 2016). CBF genes are modulated by several transcriptional factors (TFs), including ICE1 (inducer of CBF expression 1), MYB15 (Myb domain protein 15), and CAMTA3 (calmodulin-binding transcription activator 3) (Jia et al., 2016; Zhao et al., 2016). Cold stress sensing leads to a cytosolic calcium (Ca^2+^) signal (Knight et al., 1996). The activated Ca^2+^ signal is postulated to activate a MAPK (mitogen-activated protein kinase) cascade, which phosphorylate TFs, such as CAMTAs and ICE1/2 (Zhu, 2016). Recently, Ma et al. (2015) reported that the interaction between heterotrimeric guanine nucleotide-binding protein (G protein) and CHILLING TOLERANCE DIVER GENCE 1 (COLD1) activates the Ca^2+^ signal upon cold treatment in rice. However, it is not well-established how G protein triggers Ca^2+^ signal.

G protein signaling pathway is an evolutionarily conserved extracellular signal transduction. G proteins comprises three distinct subunits: alpha (Gα), beta (Gβ), and gamma (Gγ) (Tuteja and Sopory, 2008). According to the canonical paradigm, ligand-bound G protein coupled receptors (GPCRs) catalyzed exchange of GDP for GTP on the Gα actives the heterotrimer, and resulting in dissociation of the two functional elements, Gα subunit and Gβγ dimer, which mediate signal transduction by interacting with multiple downstream effectors, independently (Yuri et al., 2012). In plants, G proteins and play significant roles in many stress responses. For instance, in rice and maize, Gα functions at both cell division and cellular senescence stages of plant responses to NaCl stress (Urano et al., 2014). Microarray analysis revealed that rice Gα plays an important role in the regulation of multiple abiotic stresses, such as drought, salinity, heat, and cold (Jangam et al., 2016). Arabidopsis Gβ (AGB1) positively regulates salt tolerance by affecting the expression of genes related to proline biosynthesis, oxidative stress, ion channel, and ABA-responses (Ya-Nan et al., 2015; Swain et al., 2016). It was also reported that transcripts of *PsG*α and *PsG*β increased after heat, H_2_O_2_, and NaCl treatments in *Pisum sativum*, and over-expression of *PsG*α enhanced tolerance to salinity and heat in transgenic lines (Misra et al., 2007). However, the available set of subunits in plants are limited. In Arabidopsis genome, there is only one Gα gene (*GPA1*) and one Gβ gene (*AGB1*), while three Gγ genes (*AGG1*, *AGG2*, and *AGG3*) (Daisuke et al., 2013). And this is roughly the G protein inventory for plants; for example, rice genome contains only one canonical Gα gene (RGA1), one Gβ gene (RGB1), but five Gγ subunits (RGG1, RGG2, GS3, DEP1, and OsGGC2) (Yuri et al., 2012; Daisuke et al., 2013). With single Gα and Gβ subunits, the specificity of heterotrimer formation is thus solely provided by the Gγ proteins (Trusov et al., 2007, 2008). It is thus important to study the roles of Gγ subunits in triggering Ca^2+^ signaling for chilling tolerance in plant.

In cucumber, a typical chill-sensitive vegetable crop widely cultivated in the world, low temperatures can result in chilling injuries and lead to significant yield decreases. In the present study, we identified six Gγ proteins encoded by the cucumber genome. The transcript levels of *CsGG3.2*, encoding a type C Gγ, were up-regulated by cold treatment. *CsGG3.2* over-expressing enhanced tolerance of cucumber to chilling stress, and positively regulated the expression of *CBF* genes and their regulon, as well as activity of enzymes related to reactive oxygen species (ROS) scavenging. We conclude that the type C Gγ subunit CsGG3.2 mediates cold signal transduction in cucumber.

## Materials and Methods

### Plant Materials and Growth Conditions

Cucumber (*Cucumis sativus* L.) line 9930 donated by Huang et al. (2009) was used for gene cloning and “Xintai Mici” was used for the construction of transgenic plants. Cucumber seeds germinated in darkness at 28°C were sowed in vermiculite-peat mixture [1:1, volume/volume (V/V)] in the growth chamber under a 12 h light (350 μmol m^-2^ s^-1^) at 25°C/12 h dark at 18°C cycle. Plants with two true leaves were exposed to cold stress at 8 ± 1°C for 7 days, with 350 μmol m^-2^ s^-1^ light for 12 h every day according to Wang et al. (1999).

### Plant Transformation

To generate over-expression lines, the coding sequence of *CsGG3.2* was amplified and cloned into the *Bam*HI/*Spe*I sites of the pCAMBIA-2300 vector to obtain the Pro35S:CsGG3.2 construct. *Agrobacterium* LB4404 harboring the construct was used for cucumber transformation. The transformation was conducted according to the method of Mu et al. (2017). Briefly, cucumber seeds were rinsed in sterile deionized water for five times and placed on MS0 medium (MS plus 3% sucrose) for 2–3 days after being disinfected with 70% alcohol for 20 s followed by 3% sodium hypochlorite solution for 7 min. The basal cotyledons were then harvested and incubated with *Agrobacterium* harboring the target constructs for 15 min. The inoculated explants were then cultured on MS1 medium (MS0 medium plus 0.5 mg L^-1^ 6-Benzylaminopurine and 1 mg L^-1^ ABA) for another 2 days in the dark. The explants were transferred and incubated on MS1 medium for 15–20 days until the shoots were 1–1.5 cm long. The shoots were transferred to MS2 (MS plus 200 mg L^-1^ cefotaxime) to develop the roots. Polymerase chain reaction (PCR) was employed to confirm the integration of the construct in regenerated plants.

### Quantitative Real-Time Polymerase Chain Reaction (qRT-PCR)

Total RNA was isolated using RNA prep pure Plant Kit (TANGEN) and first-strand cDNA was synthesized using Fast Quant RT Kit (TANGEN) according to the manufacturer’s instructions. PCR was then carried out using the gene-specific primers listed in Supplementary Table S1 and Super Real PreMix Plus (SYBR Green) Kit (TANGEN) with an Mx3000p Real-time PCR System (Agilent, Stratagene) according to the manufacturer’s instructions. Three biological replicates were included for each sample. The 2^-ΔΔCt^ method was used, and the relative expression levels were normalized to *Actin*.

### Biochemical Analysis Assays

Oxygen free radical (OFR), H_2_O_2_, and malondialdehyde (MDA) content, activity of SOD, catalase (CAT), peroxidase (POD), and glutathione reductase (GR) were determined using assay kits (COMINBIO) with a UV-1800 Spectrophotometer (SHIMADZU) according to the manufacturer’s instructions. OFR content was assayed based on the detection of the absorbance of product at 530 nm in the reaction system. H_2_O_2_ content was assayed based on the titanium superoxide synthesis method. MDA content was assayed based on the thiobarbituric acid-reactive substance assay. The SOD activity was determined based on the inhibition of formazan synthesis method. The CAT activity was determined based on the decomposition of H_2_O_2_ method. The POD activity was assayed based on the detection of the absorbance of product at 470 nm in the reaction system. The GR activity was determined based on the NADPH consumption method.

### Assessment of Chilling Tolerance of Cucumber

T1 transgenic lines and control plants with/without cold-acclimated were exposed to 4 ± 1°C for 4 days, with 350 μmol m^-2^ s^-1^ light for 12 h every day. Chilling injury (CI) was indexed following Liu et al. (2010). The severity of the symptoms was assessed visually in a four-stage scale: (1) no injury; (2) slight; (3) moderate; (4) extensive. The average extent of cold damage was expressed as CI index, which was calculated using the following formula: CI index (%) = [Σ(CI level) × (number of seedlings at the CI level)/(total number of seedlings) × 4] × 100. Cold acclimation was performed at continuous 8 ± 1°C with 12 h photoperiod (350 μmol m^-2^ s^-1^) for 2 days.

### Data Analyses

The results were analyzed using GraphPad Prism 6.0 (GraphPad Software) and Data Processing System (DPS) 7.05 (Tang and Zhang, 2013). Three biological replicates were included for each experiment. Data are presented as mean values ± SE (*n* = 3). The analyses of significant differences (*p* < 0.05/*p* < 0.01) were measured using least significant difference (LSD) test.

## Results

### The Cucumber Proteome Contains Six Heterotrimeric G Protein Gγ Subunits

**FIGURE 1:**
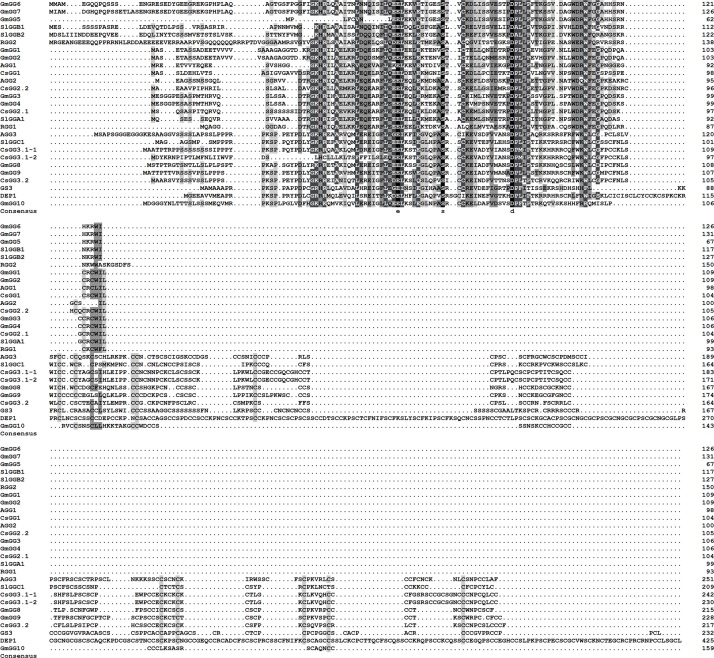
Multiple sequence alignments of CsGG with Gγ amino acids from other species. CsGG and its homologs were aligned using CLUSTAL X. Conserved regions between single classes or the whole family are shown in white text on black, with conserved substitutions shaded in gray. AGG1: *Arabidopsis thaliana* Q9FDX9 (Uniprot accession No.); AGG2: *Arabidopsis thaliana* Q93V47; AGG3: *Arabidopsis thaliana* Q6AWT8; RGG1: *Oryza sativa* subsp. *Japonica* Q75WU1; RGG2: *Oryza sativa* subsp. *Japonica* Q6YXX9; GS3: *Oryza sativa* subsp. *Japonica* C6L686; DEP1: *Oryza sativa* subsp. *Japonica* Q67UU9; GmGγ1: *Glycine max* L. Glyma10g03610 (Soybean Genome accession No.); GmGγ2: *Glycine max* L. Glyma02g16190; GmGγ3: *Glycine max* L. Glyma20g35415; GmGγ4: *Glycine max* L. Glyma10g32214; GmGγ5: *Glycine max* L. Glyma11g18050; GmGγ6: *Glycine max* L. Glyma14g17060; GmGγ7: *Glycine max* L. Glyma17g29590; GmGγ8: *Glycine max* L. Glyma15g19631; GmGγ9 : *Glycine max* L. Glyma17g05640; GmGγ10 : *Glycine max* L. Glyma.07G040200; SlGGA1: *Solanum lycopersicum* Solyc09g082940.2.1 [Tomato Genome (cv Heinz; ITAG release 2.40) accession No.]; SlGGB1: *Solanum lycopersicum* Solyc12g096270.1.1; SlGGB2: *Solanum lycopersicum* Solyc08g005950.2.1; SlGGC1: *Solanum lycopersicum* Solyc07g041980.2.1.

**FIGURE 2:**
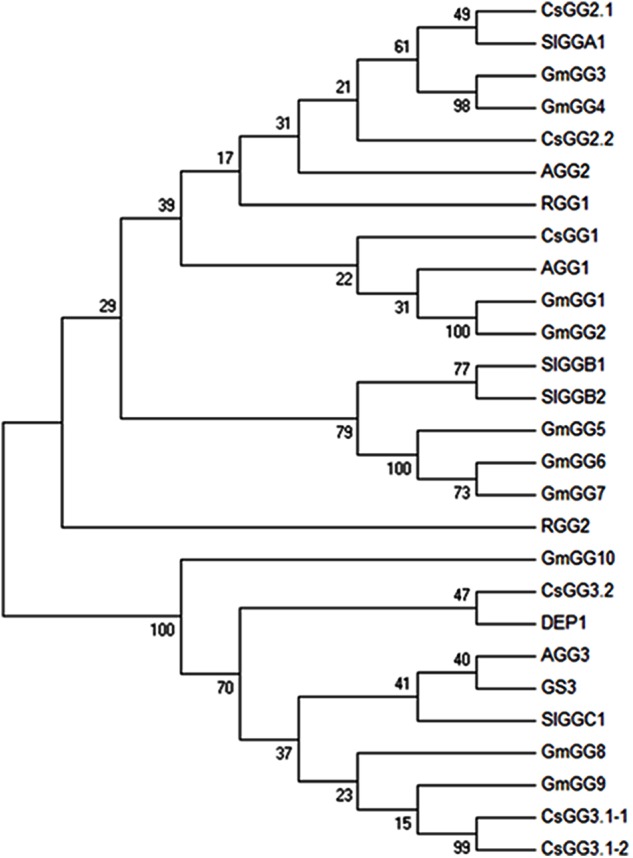
Phylogenetic analysis of CsGGs and other Gγ proteins in plants. Tree was constructed using neighbor-joining method with 1000 bootstrap replicates.

BLAST searches of the cucumber genome^1^ using Arabidopsis Gγ subunits as queries identified five Gγ-like genes. We named these genes *CsGG1* (*Csa2G228360*), *CsGG2.1* (*Csa2G215490*), *CsGG2.2* (*Csa3G144190*), *CsGG3.1* (*Csa2G000110*), and *CsGG3.2* (*Csa1G597050*). *CsGG3.1* alternative splicing produces two protein variants (CsGG3.1-1 and CsGG3.1-2). The six cucumber Gγ homologs were divided into two classes based on amino acid sequence alignments (**Figure 1**) and phylogenic methods (**Figure 2**). CsGG1, CsGG2.1, and CsGG2.2 belonged to the previously described type A, while CsGG3.1-1, CsGG3.1-2, and CsGG3.2 belonged to type C (Trusov et al., 2012).

### *CsGG3.2* Exhibited a Cold Inductive Expression Pattern

**FIGURE 3:**
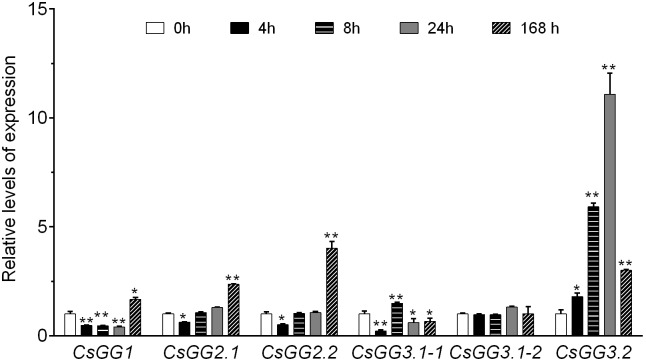
Relative expression of *CsGG* genes in wild type (WT) cucumber under cold stress. RNA was extracted and the expression levels of the *CsGG* genes were analyzed by qRT-PCR. Data shown are averages ± SE (*n* = 3). Three biological replicates were included for each experiment and 10 seedlings were included for each line. Significant differences from plants at 0 h are indicated by asterisks (^∗^*p* < 0.05 and ^∗∗^*p* < 0.01).

To investigate the cold responsive characteristic of *CsGG* genes, the expression patterns of these genes in leaves were detected by quantitative real-time polymerase chain reaction (qRT-PCR). As shown in **Figure 3**, *CsGG1* transcript decreased from 0 to 24 h, followed by an increase at 168 h. *CsGG2.1* and *CsGG2.2* showed similar expression profiles, with transcript levels decreased at 4 h, while increased apparently at 168 h. *CsGG3.1-1* showed relatively low transcript levels at most of the time, while the transcript of *CsGG3.1-2* showed no response to cold treatment. Among the five selected time points, the transcript levels of *CsGG3.2* climbed to the peak at 24 h and descended to a lower level at 168 h. These results demonstrate that *CsGG3.2* might be involved in cold tolerance in cucumber.

### Over-Expression of *CsGG3.2* Enhanced the Cold Tolerance of Transgenic Cucumbers

**FIGURE 4:**
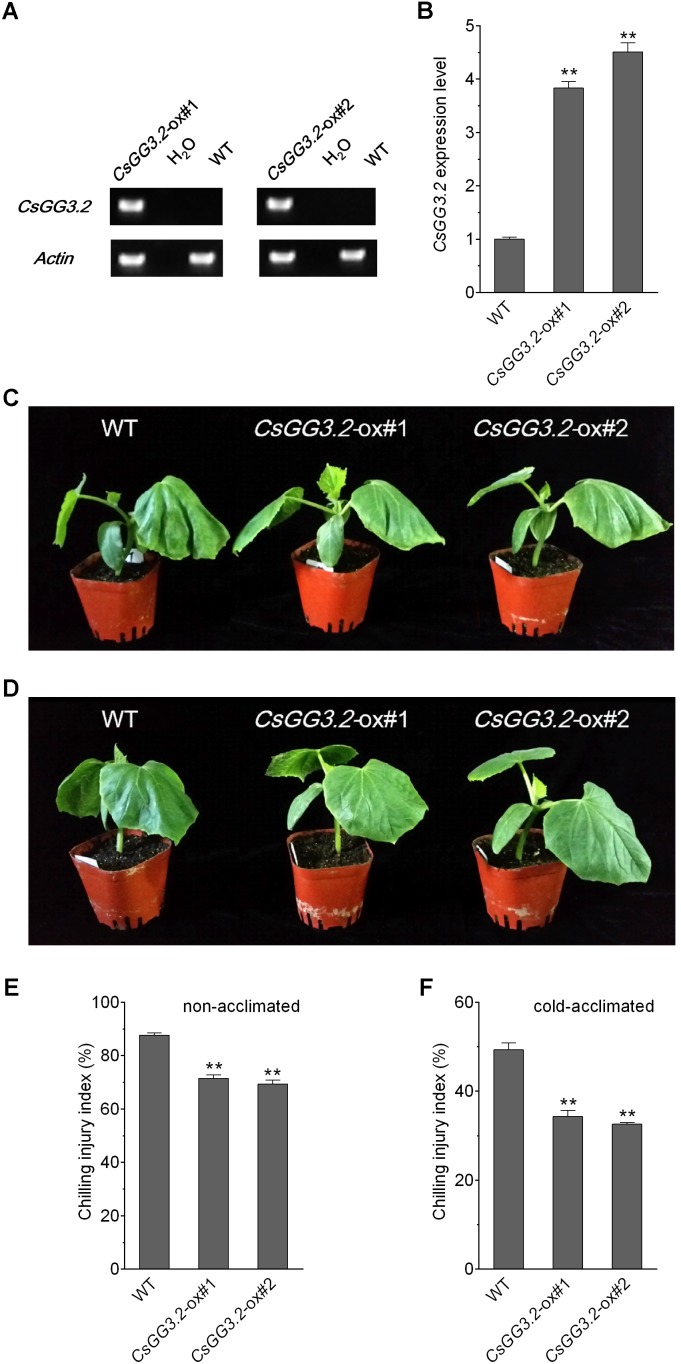
CsGG3.2 enhances cucumber chilling tolerance. **(A)** PCR analysis of the *CsGG3.2* over expressing T1-transgenic lines along with WT, and negative control (H_2_O). **(B)** Expression level of the *CsGG3.2* mRNA in *CsGG3.2* over expressing T1-transgenic lines grown for 7 days. Chilling sensitivity of *CsGG3.2* over expressing T1-transgenic and WT plants non-acclimated **(C)** and cold-acclimated **(D)**. Chilling injury (CI) index of *CsGG3.2* over expressing T1-transgenic and WT plants non-acclimated **(E)** and cold-acclimated **(F)**. The data represent means ± SE (*n* = 3) of three independent replicates with 10 seedlings (20 seedlings for chilling tolerance assessment) per line per replicate. The asterisks indicate a significant difference between WT and *CsGG3.2* over-expressing plants (^∗∗^*p* < 0.01).

To investigate whether the *CsGG3.2* function in chilling tolerance, transgenic cucumber plants over-expressing *CsGG3.2* were constructed. Two lines (*CsGG3.2*-ox#1 and *CsGG3.2*-ox#2) with *CsGG3.2* expression level about four fold higher compared with wild type (WT) cucumber (**Figures 4A,B**) were exposed to chilling stress to examine the chilling tolerance before and after cold acclimation. For non-acclimated plants, WT plants exhibited severe injury in their cotyledons, and even the first true leaves. Two transgenic lines *CsGG3.2*-ox#1 and *CsGG3.2*-ox#2 showed lighter injuries (**Figure 4C**), and their CI indices significantly lower than WT, and were decreased by 18 and 21%, respectively (**Figure 4E**). For cold-acclimated plants, WT plants exhibited obvious injury in their cotyledons and first true leaves. Transgenic plants showed slight or no injury, and their CI indices were decreased by 30 and 34%, respectively (**Figures 4D,F**) compared with WT. These results suggest that the expression of *CsGG3.2* enhanced the chilling tolerance of transgenic cucumber plants.

### Over-Expression of *CsGG3.2* Enhanced *CBF* and *COR* Genes Transcripts Upon Exposure to Cold

**FIGURE 5:**
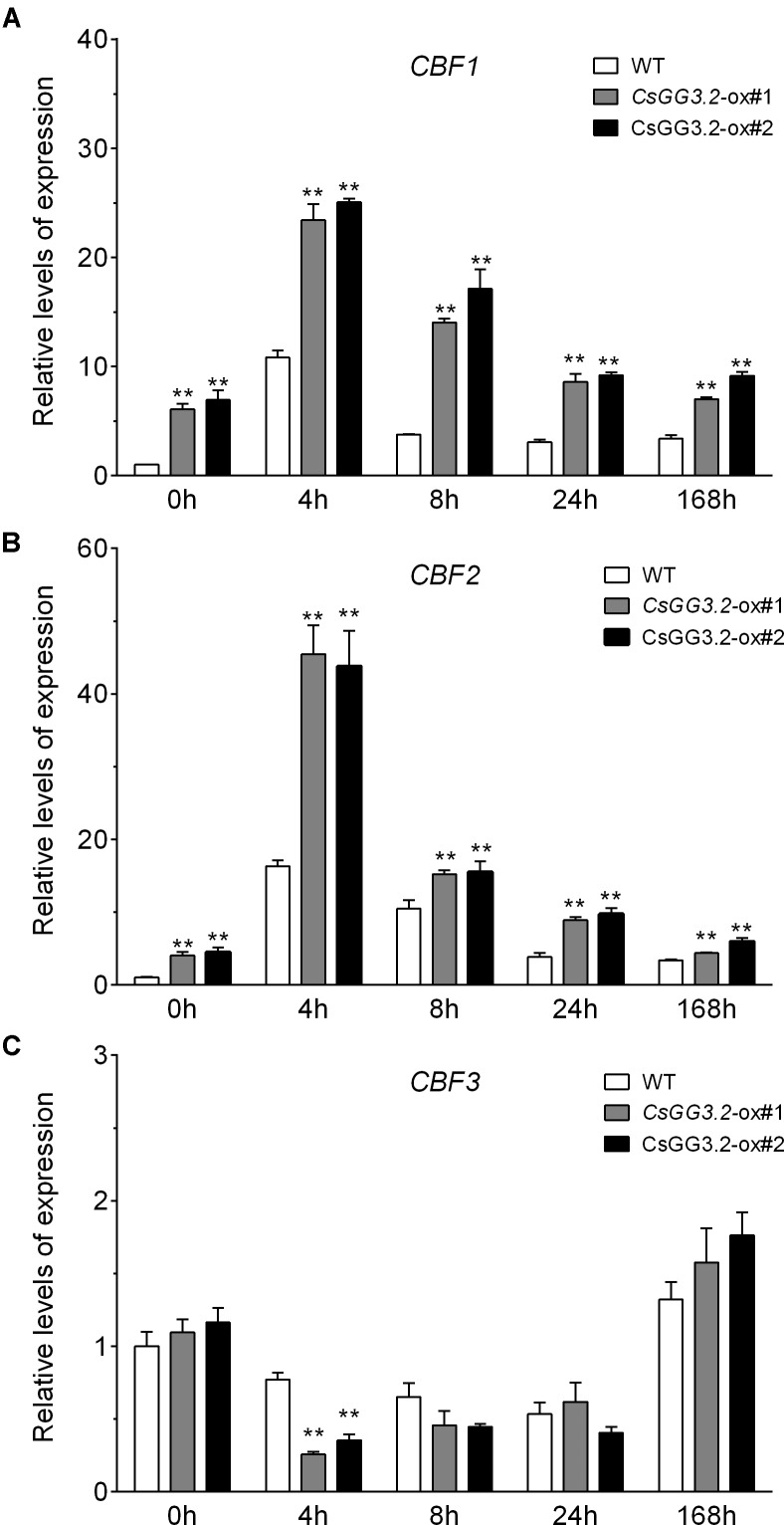
Relative gene expression of cucumber homologs *CBF* genes in *CsGG3.2* over expressing T1-transgenic and WT cucumber plants under cold stress. Sequence data of *CsCBF* genes can be found in the cucumber genome database under the following accession numbers: *CsCBF1*, Csa5G174570 **(A)**; *CsCBF2*, Csa3G180260 **(B)**; *CsCBF3*, Csa3G751440 **(C)**. Data shown are averages ± SE (*n* = 3). Three biological replicates were included for each experiment and 10 seedlings were included for each line. The asterisks indicate a significant difference between WT and *CsGG3.2* over-expressing plants (^∗∗^*p* < 0.01).

**FIGURE 6:**
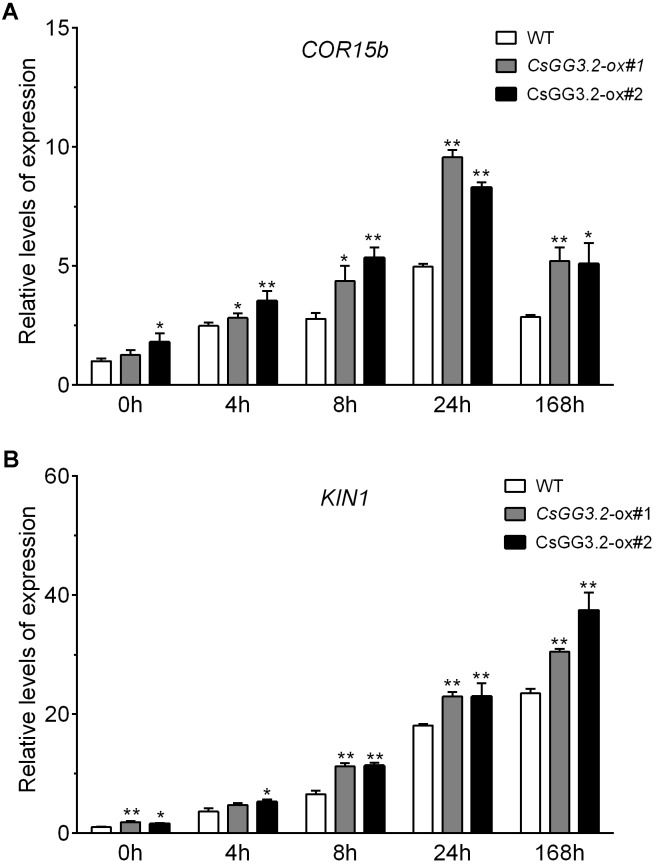
Relative gene expression of cucumber homologs *COR15b* **(A)**, and *KIN1* **(B)** in *CsGG3.2* over-expressing T1-transgenic and WT plants under cold stress. Sequence data of *CsCOR15b* and *CsKIN1* can be found in the cucumber genome database under the accession number Csa1G074950 and Csa5G165870 respectively. Data shown are averages ± SE (*n* = 3). Three biological replicates were included for each experiment and 10 seedlings were included for each line. The asterisks indicate a significant difference between WT and *CsGG3.2* over-expressing plants (^∗^*p* < 0.05 and ^∗∗^*p* < 0.01).

Induction of *CBF* genes is important for chilling stress tolerance (Zhu et al., 2007). Therefore, we investigated the expression levels of *CBF* genes and their downstream target genes, *COR15b* and *KIN1* in *CsGG3.2* over-expressing transgenic and WT cucumber plants during cold acclimation. Cucumber *CBF* and *COR* genes were obtained by database searching using protein sequences of Arabidopsis CBFs, COR15b, and KIN1 as queries. *Csa5G174570* and *Csa3G751440* corresponded to the published *CsCBF1* (GenBank, DQ776899) and *CsCBF3* (GenBank, JQ900769), respectively. *Csa3G180260* coding a protein sharing high identities to AtCBF2, was named *CsCBF2. Csa1G074950* and *Csa5G165870* coding late embryogenesis abundant (LEA) proteins were designated as *CsCOR15b* and *CsKIN1*, respectively. The transcript levels for *CsCBF1* and *CsCBF2* increased within 4 h of plants being exposed to cold stress. The levels of *CsCBF1* and *CsCBF2* expression was significantly higher in *CsGG3.2*ox plants compared with WT plants (**Figures 5A,B**). Levels of *CsCBF3* transcript were relatively lower compared to that of *CsCBF1* and *CsCBF2. CsGG3.2* over-expressing significantly reduced the transcript level of *CsCBF3*, but had no effect at other time points (**Figure 5C**). Cold increases the *CsCOR15b* and *CsKIN1*, two known *CBF*-inducible COR genes, expression in both WT and *CsGG3.2*ox plants. And their expression levels were significantly up-regulated by *CsGG3.2* over-expressing (**Figure 6**).

### Analysis of Antioxidant Enzymes Activity and Response of ROS and Malondialdehyde (MDA) in Transgenic Plants

**FIGURE 7:**
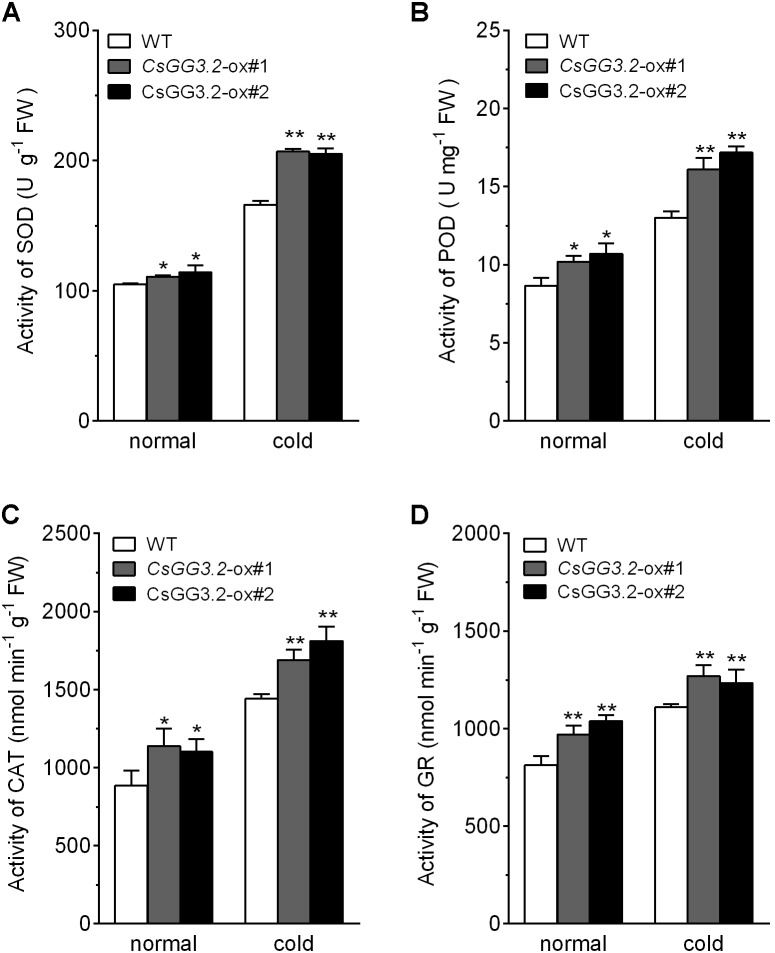
Antioxidant activities of *CsGG3.2* over-expressing T1-transgenic and WT plants under cold stress. **(A)** Superoxide dismutase (SOD), one unit of enzyme activity defined as 50% formazan synthesis inhibited in the xanthine oxidation coupling reaction solution. **(B)** Peroxidase (POD), one unit of enzyme activity defined as the absorbency at 470 nm increased by 0.01 in 1 ml reaction solution min^-1^ per gram tissue. **(C)** Catalase (CAT) activity, one unit of enzyme activity defined as 1 nmol H_2_O_2_ oxidized min^-1^ per gram tissue. **(D)** Glutathione reductase (GR), one unit of enzyme activity defined as 1 nmol NADPH oxidized min^-1^ per gram tissue. Data shown are averages ± SE (*n* = 3). Three biological replicates were included for each experiment and 10 seedlings were included for each line. The asterisks indicate a significant difference between WT and *CsGG3.2* over-expressing plants (^∗^*p* < 0.05 and ^∗∗^*p* < 0.01).

**FIGURE 8:**
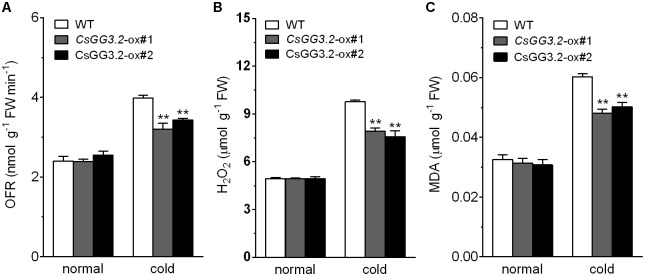
Biochemical analysis of *CsGG3.2* over-expressing T1-transgenic and WT plants under cold stress. **(A)** Oxygen free radical (OFR) productive rate per gram tissue. **(B)** Hydrogen peroxide (H_2_O_2_) content. **(C)** Lipid peroxidation expressed in terms of malondialdehyde (MDA) content. Data shown are averages ± SE (*n* = 3). Three biological replicates were included for each experiment and 10 seedlings were included for each line. The asterisks indicate a significant difference between WT and *CsGG3.2* over-expressing plants (^∗∗^*p* < 0.01).

The changes induced by cold in the activities of antioxidant enzymes and production of OFR, H_2_O_2_, and malondialdehyde (MDA) in transgenic lines were compared with WT plants. Over-expression of *CsGG3.2* resulted in increased enzymatic activities of SOD, POD, CAT, and GR in transgenic plants under both normal and cold conditions (**Figures 7A–D**). And this resulted in decreased accumulation of OFR and H_2_O_2_ in the transgenic plant in stressful environment compared with WT plants (**Figures 8A,B**). The increased detoxification of ROS led to reduced membrane lipid peroxidation, i.e., MDA production. MDA level was low (about 0.031 μmol g^-1^ FW) under normal conditions in transgenic and WT plants. MDA content in WT plants increased to 0.060 μmol g^-1^ FW under chilling stress, whereas with a lower increase, to only 0.048 μmol g^-1^ FW and 0.050 μmol g^-1^ FW respectively, was seen in the transgenic plants (**Figure 8C**).

## Discussion

Plant Gγ subunits are involved in a wide range of developmental and physiological processes, and have a high potential for crop improvements (Botella, 2012). In rice, *DEP1* and *GS3* are major quantitative trait loci for controlling seed size and panicle branching (Botella, 2012); *RGG1* and *RGG2* are up-regulated upon salinity, harsh temperature, and ABA treatments (Yadav et al., 2012). Arabidopsis *AGG1* and *AGG2* are reported to be involved in osmotic stress and root development, and *AGG3* is also found to be involved in regulation of organ size and stress response (Chakravorty et al., 2011; Li et al., 2012a,b; Thung et al., 2013). Similarly, the soybean GγIII subunit plays a role in ABA-dependent lateral root development (Choudhury and Pandey, 2013). Tomato *SlGGB1* also mediates auxin and ABA signaling (Subramaniam et al., 2016). Here, we demonstrated that over-expression of *CsGG3.2* promotes tolerance of cucumber seedlings to chilling stress. Moreover, multiple mechanisms appear to contribute to this increase in freezing tolerance, including alterations in gene expression associated with cold acclimation and the activation of antioxidative enzymes.

Chilling tolerance of plants was enhanced by cold acclimation. DREB1/CBFs are transcription factors regulating the expression of more than 100 COLD RESPONSIVE (COR) genes, and thus important for cold acclimation and chilling tolerance (Chinnusamy et al., 2007; Park et al., 2015). Constitutive expression of any one of the *CBF* genes in transgenic plants gives rise to strong constitutive expression of the *COR* genes and hence increased freezing tolerance in plants (Jagloottosen et al., 1998; Kasuga et al., 1999; Gilmour et al., 2004; Nakashima, 2006). Expression of *CsCBF1* and *CsCBF2* were upregulated in *CsGG3.2*ox plants (**Figures 5A,B**), supporting the proposed role of *CsGG3* in promoting chilling tolerance. However, transcription levels of *CsCBF3* were lower than that of *CsCBF1* and *CsCBF2* in both WT and transgenic plants (**Figure 5C**). This results may be due to the negative interaction between *CBF* genes, which is important for transient and tightly controlling their expression (Knight and Knight, 2012).

The expression of *CBF* genes activate many downstream genes that enhance plants chilling and freezing tolerance (Park et al., 2015). Many CBF-inducible genes have been cloned and characterized from Arabidopsis and other species. *COR15a* encodes a chloroplast-targeted polypeptide, which functions as cryoprotective protein preventing the formation of hexagonal II-phase lipids in the chloroplast stroma (Nakayama et al., 2007). The homolog of *AtCOR15a*, *AtCOR15b* has transcript profiles similar to that of *AtCOR15a* under cold stress (Wilhelm and Thomashow, 1993). And the transcript level of *CbCOR15b* from shepherd’s purse (*Capsella bursa-pastoris*) was significantly upregulated under cold treatment, and over-expression of *CbCOR15b* enhanced cold tolerance in transgenic tobacco plants (Wu et al., 2012). *KIN1* is another CBF downstream target gene from Arabidopsis encoding a 6.5 KDa polypeptide similarity to the type I fish antifreeze proteins. In Cucurbita crops, watermelon and pumpkin, transcripts of *CmCOR15b*, *ClKIN1*, and *CmKIN1* significantly increased during cold stress condition, suggesting that they could contribute to the cold tolerance (Kang et al., 2009). Here, we showed that the transcript levels of cucumber *CsCOR15b* and Cs*KIN1* increased dramatically in response to low temperature (**Figure 6**), which are consistent with that previously found in watermelon and pumpkin (Kang et al., 2009). Meanwhile, expression of *CsCOR15b* and Cs*KIN1* was upregulated by *CsGG3.2* over-expressing (**Figure 6**), suggesting CsGG3.2 facilitates induction of *COR* genes expression, and cold tolerance.

Reactive oxygen species were produced and accumulated under cold stress, which damaged cell and yielded MDA (Achard et al., 2008; Swain et al., 2016). Antioxidant enzymes are important components of the ROS scavenging system in the plant cell, thus play a significant role in plant cold tolerance (Ahmad et al., 2010; Liu et al., 2010). It has been reported that activities of antioxidant enzymes in plants are increased under low temperature stress, which might be due to the upregulation of corresponding genes (Baek and Skinner, 2003; Soltész et al., 2011). In present study, the activity of SOD, POD, CAT, and GR enzymes in WT plants increased under cold condition, but the rates of increase were higher in transgenic plants (**Figure 7**). The increased detoxification of ROS led to reduced membrane lipid peroxidation, and could increase the chilling tolerance of transgenic plants. These results are in agreement with the previous studies where a decreased level of ROS production under cold stress has been reported in *ICE1*-ox cucumber (Liu et al., 2010).

Heterotrimeric G proteins play important roles in stress responses by interacting with Ca^2+^ channels in animals (Wang and Chong, 2010). Recently, plant G protein signaling found to be involved in cold signal transduction: activation of G protein by COLD1, triggered Ca^2+^ influx upon cold treatment, leading to a cytosolic Ca^2+^ signal (Ma et al., 2015). Ca^2+^ signal is postulated to activate a MAP kinase cascade, which then phosphorylate cold responsive TFs such as CAMTAs and ICE1/2 (Zhu, 2016). The exact mechanism of G protein-mediated plant cold response is not known yet. Here, our findings showed that CsGG3.2 positively regulates the expression of *CBF* genes and their regulon, as well as the activities of antioxidant enzymes, which lead to cold stress tolerance. The results suggest a critical role of CsGG3.2 in cucumber cold response, which will help in understanding the G protein-mediated cold signal transduction in plants.

## Author Contributions

XY, YaL, LB, and CH: conceived and designed the experiments. LB and YuL: performed the experiments. LB: analyzed the data. LB, YM, and YY: contributed reagents/materials/analysis tools. LB, YuL, and AA: wrote the paper.

## Conflict of Interest Statement

The authors declare that the research was conducted in the absence of any commercial or financial relationships that could be construed as a potential conflict of interest.

## Footnotes

1 http://www.icugi.org
