# Supplementary material for: Heterotrimeric G-Protein γ Subunit CsGG3.2 Positively Regulates the Expression of CBF Genes and Chilling Tolerance in Cucumber
Source: Front Plant Sci. 2018 Apr 17;9:488. doi: 10.3389/fpls.2018.00488 (PMC5913349; doi:10.3389/fpls.2018.00488)
Supplement: Supplementary file 1 [file Table_1.DOCX]

**SUPPLEMENTARY MATERIAL**

**Supplementary Table S1.** **Primers used in this study**

| Primer Name | Primer Sequence (5’ to 3’) | Experiment |
| --- | --- | --- |
| CsGG3.2-BamHI-2300-S | CGCGGATCCATGGCTGCTCGATCGGTTTACT | Plant Transformation |
| CsGG3.2-SpeI-2300-A | CTAGACTAGTAAAGCAACAACATAAAGAGCAAGGG |  |
| CsGG1-qPCR-S | AAGCGTCTGGAGCAGGAACT | qRT-PCR |
| CsGG1-qPCR-A | AACCCACGATGGATTTACAACA |  |
| CsGG2.1-qPCR-S | AGTCTGGAGGAAGAACTGGAACA |  |
| CsGG2.1-qPCR-A | TGGATTAGTGGGAGAAGAGGGT |  |
| CsGG2.2-qPCR-S | AGGATGCTCGCTGAAATGAAG |  |
| CsGG2.2-qPCR-A | CTGTCTATGCTGCCCAATAACTC |  |
| CsGG3.1-1-qPCR-S | TATTTCCACTCGCCCACCTT |  |
| CsGG3.1-1-qPCR-A | GCTTTCCTGACAAATCTGGGT |  |
| CsGG3.1-2-qPCR-S | TGAGAAATCCCATTCCCACAT |  |
| CsGG3.1-2-qPCR-A | CTGAGTTGGCGGTCACATAGTC |  |
| CsGG3.2-qPCR-S | AGGTGTTGTAGTATGCCGAAGTGT |  |
| CsGG3.2-qPCR-A | GCGTGGGATTGAAGGTAGTGAT |  |
| CsCBF1-qPCR-S | GCGACTGAGGAATTCTGGTAA |  |
| CsCBF1-qPCR-A | GGGAAAGTTCCAAGCCAAATC |  |
| CsCBF2-qPCR-S | TTATGTTAGCCGCCAGTAATCC |  |
| CsCBF2-qPCR-A | CTCCTCCTGACTCCTCTGTAAA |  |
| CsCBF3-qPCR-S | CTCAACCAACGACCACCT |  |
| CsCBF3-qPCR-A | CGCAAACCCATTTACCAT |  |
| CsCOR15b-qPCR-S | GAAGGCGAAGGAAGGAACAA |  |
| CsCOR15b-qPCR-A | TCGCCATCTCCTTTGTCTTATC |  |
| CsKIN1-qPCR-S | CCAGAACATGAGCTACCAGATT |  |
| CsKIN1-qPCR-A | CTGCTGTCCTCCTTCTTGTATT |  |
| CsActin-qPCR-S | TTCTGGTGATGGTGTGAGTC |  |
| CsActin-qPCR-A | GGCAGTGGTGGTGAACATG |  |
| CsGG3.2-PCR-S | AAGGTGTTGTAGTATGCCGAAGT | RT-PCR |
| CsGG3.2-PCR-A | ATGCCGAGAGTGATCCCG |  |
| CsActin-PCR-S | CCTCAATCCTAAAGCCAACAGA |  |
| CsActin-PCR-A | ATAGACCCTCCAATCCAAACACT |  |
